# Intravenous Paracetamol for Perioperative Shivering Prophylaxis in Patients Undergoing Spinal Anesthesia: A Prospective, Randomized, Double‐Blind Controlled Trial

**DOI:** 10.1155/anrp/6133770

**Published:** 2026-11-10

**Authors:** Thitinuch Ruenhunsa, Saranyoo Nonphiaraj, Donlaya Srikajonkiat, Kanya Panichakul, Sarinya Chanthawong, Chanapat Charoensuk, Peerapong Sangsungnern, Pasit Surakamhaeng

**Affiliations:** ^1^ Department of Anesthesiology Faculty of Medicine Khon Kaen University 123 Mittraphap Road, Nai Mueang, Mueang Khon Kaen Khon Kaen 40002 Thailand kku.ac.th

**Keywords:** paracetamol, perioperative shivering, pethidine, prophylaxis, spinal anesthesia

## Abstract

**Background:**

Perioperative shivering (POS) is a common, distressing complication of spinal anesthesia (SA), but a standard prevention has not been identified. This study aimed to determine whether intravenous (IV) paracetamol prevents POS in patients undergoing surgery under SA.

**Methods:**

In this prospective, randomized, double‐blind controlled trial, 92 patients scheduled for surgery under SA received IV paracetamol 15 mg/kg (*n* = 46) or an equal volume of saline (*n* = 46) over 15 min before SA. The primary outcome was the overall incidence of POS. The primary analysis was per‐protocol (PP) (*n* = 86; paracetamol 42; control 44) after exclusion of 6 patients postrandomization. Post hoc best‐case and worst‐case sensitivity analyses including all 92 patients were also performed. Secondary outcomes included shivering score, shivering duration, rescue pethidine requirements, temperature changes, and adverse events.

**Results:**

Baseline and intraoperative characteristics were comparable between groups. In the PP analysis, POS incidence was lower with paracetamol than control (26.2% vs. 50.0%; *p* = 0.023). This was not robust to missing‐data assumptions: the best‐case analysis remained significant (23.9% vs. 52.2%; *p* = 0.005), but the worst‐case analysis did not (32.6% vs. 47.8%; *p* = 0.137), and the fragility index was 2. Intraoperative shivering occurred in 4.8% vs. 22.7% (*p* = 0.016). Paracetamol also demonstrated lower highest shivering score (*p* = 0.039), shorter duration of shivering (*p* = 0.031), and less rescue pethidine use (*p* = 0.019). There were no statistically significant differences in temperature changes, hypothermia, or nausea and vomiting between groups.

**Conclusions:**

In the PP analysis, prophylactic IV paracetamol was associated with a lower incidence, severity, and duration of POS and a lower need for rescue pethidine use. However, the primary finding was not robust under worst‐case assumptions (fragility index 2); larger confirmatory trials are warranted.

**Trial Registration:** Thai Clinical Trials Registry: TCTR20210209004.

## 1. Introduction

Perioperative shivering (POS) is a frequent and distressing complication following spinal anesthesia (SA), with an incidence ranging from 40% to 64% [1, 2]. Shivering is fundamentally a physiological response; it occurs when core body temperature drops below the thermoregulatory set point governed by the hypothalamus [3]. In the context of neuraxial blockade, SA profoundly impairs temperature control primarily by blocking sympathetic pathways, leading to excessive redistribution of core heat to peripheral tissues and to intraoperative environmental heat loss [4–6].

Beyond subjective patient discomfort, POS poses significant physiological hazards, particularly in high‐risk populations. Severe shivering exponentially increases metabolic demand and oxygen consumption, stimulates catecholamine release, and elevates cardiac output, which may precipitate myocardial ischemia [7, 8]. Furthermore, intense tremors can interfere with continuous physiological monitoring—such as electrocardiography, noninvasive blood pressure, and pulse oximetry—potentially masking intraoperative complications and subsequently prolonging recovery time in the postanesthesia care unit (PACU) [9]. Therefore, implementing effective preventative strategies against POS during continuous SA is a crucial aspect of perioperative care.

Current standard practice advocates for multimodal approaches, combining nonpharmacological measures (e.g., active warming strategies) with pharmacological agents [6, 8–11]. Various drugs, including opioids, *α*_2_‐agonists (e.g., dexmedetomidine), and adjuvants, have been investigated for their antishivering effects during the perioperative period [7, 12–16]. However, many conventional agents are often limited by unfavorable side effects such as pronounced sedation, respiratory depression, or hemodynamic shifts [7, 14, 15].

Intravenous (IV) paracetamol offers an alternative due to its excellent safety profile and minimal side effects. Paracetamol acts centrally by inhibiting prostaglandin synthesis in the thermoregulatory center, effectively lowering the hypothalamic set point and inhibiting shivering [17]. While prophylactic IV paracetamol has shown significant antishivering efficacy in patients undergoing general anesthesia [12, 18–20] and in obstetric populations undergoing cesarean sections under SA [21], however, current evidence for paracetamol’s role in preventing POS during the broader cohort of patients undergoing procedures under SA remains limited. Therefore, the aim of this study was to evaluate the efficacy of IV paracetamol in preventing POS in patients undergoing surgery under SA.

## 2. Materials and Methods

### 2.1. Study Design and Ethics

This prospective, double‐blinded, placebo‐controlled clinical trial was conducted at Srinagarind Hospital, Faculty of Medicine, Khon Kaen University, Thailand, between February 2021 and June 2021. The trial was approved by the Center for Ethics in Human Research, Khon Kaen University (Reference No: HE631446). This study was prospectively registered with the Thai Clinical Trials Registry (TCTR20210209004; registered 9 February 2021) prior to enrollment of the first participant (15 February 2021). The trial was conducted in accordance with the Declaration of Helsinki and Good Clinical Practice guidelines. Written informed consent was obtained from every participant prior to enrollment.

### 2.2. Participants

Adult patients aged 18–70 years old with American Society of Anesthesiologists (ASA) Physical Status I–III scheduled for elective lower abdominal and lower extremities surgery under SA with expected duration of surgery less than 4 h were accessed for eligibility. The exclusion criteria were: refusal to participate; pregnancy; liver disease (e.g. cirrhosis, AST or ALT increased more than 3‐fold of upper limit of normal, and history of alcohol dependence); renal impairment with estimated glomerular filtration rate less than 30 mL/min; baseline body temperature above 37.8°C or below 36°C; history of paracetamol, tramadol, or pethidine use within 6 h before surgery; chronic use of NSAIDs more than 2 weeks; urological procedure; neurological disease (e.g. Parkinson’s disease, seizure disorders, or other conditions that may affect thermoregulation or shivering assessment); or allergy to paracetamol.

### 2.3. Randomization and Blinding

Participants were randomly assigned to the paracetamol group or the control group using a computer‐generated, blocked randomization technique (block size of 4). Allocation sequences were concealed in sequentially numbered, sealed, opaque envelopes. A single, independent study member—who was not involved in patient care or outcome assessment—was responsible for opening the envelope for each participant after transfer to the operating room (OR) and subsequently preparing the identical syringes containing either the study medication (paracetamol) or the control solution (normal saline), according to the revealed allocation. This procedure ensured that only this independent individual had knowledge of the group assignment. Participants and all research and clinical staff involved in patient care and outcome assessment remained blinded to group allocation throughout the study.

### 2.4. Intervention

The OR temperature was maintained at 20°C–22°C, and the PACU temperature was maintained at 22°C–24°C. Upon arrival in the OR, body temperature was measured using an infrared tympanic membrane thermometer (TaiDoc, model TD‐1261). To minimize measurement error, the same ear was used for each patient throughout the perioperative period. Tympanic thermometry was selected as a noninvasive, reliable surrogate for core temperature that is well tolerated in awake, nonintubated patients under SA. Patients were randomized to receive either IV paracetamol (15 mg/kg, maximum 1 g) or an equal volume of normal saline as placebo, prepared in identical 50‐mL syringes by an independent investigator not involved in patient care. The assigned solution was administered intravenously over 15 min, concurrently with loading of 5–10 mL/kg of warmed isotonic crystalloid solution, prior to SA. The SA was performed with a 27‐gauge Quincke spinal needle, using 2.5–3.5 mL of 0.5% hyperbaric bupivacaine. To standardize thermal management, all IV fluids were prewarmed to 37°C, and all patients received forced‐air warming at 38°C with upper‐body blanket coverage throughout surgery.

### 2.5. Perioperative Monitoring and Management

POS was assessed using the Bedside Shivering Assessment Scale (BSAS). Although initially developed for targeted temperature management, the BSAS was selected for this trial due to its standardized evaluation method (combining observation and muscle palpation) and its established high interrater reliability across diverse healthcare providers (*κ* = 0.66 for clinically significant shivering) [22, 23]. Furthermore, its 4‐level scoring criteria (0 = no shivering observed; 1 = shivering visible only in the neck/thorax; 2 = shivering involving gross movements of the upper extremities in addition to neck/thorax; and 3 = shivering involving gross movements of the trunk and both upper and lower extremities) directly align with the grading tiers of traditional anesthetic scales such as the Crossley and Mahajan scale.

Shivering was defined as a score ≥ 1. Hypothermia was defined as a body temperature below 36°C. Shivering score and body temperature were recorded at 15‐min intervals intraoperatively and postoperatively in the PACU. If the POS was developed, rescue pethidine (0.5 mg/kg IV) was administered; a repeat dose was permitted 10 min later if shivering persisted. Intraoperative and postoperative morphine were administered on an on‐demand basis, guided by patient‐reported pain scores. The total dose of intraoperative morphine administered was recorded.

Postoperatively in the PACU, patients were covered with a cotton blanket and monitored for 1 h. On detection of hypothermia (body temperature below 36°C) or shivering (score ≥ 1), forced‐air warming was initiated. If shivering persisted after 5 min of warming, pethidine was administered per the intraoperative protocol. The frequency and total doses of pethidine, intraoperative morphine used, and incidence of adverse effects (hypothermia, nausea and vomiting, and allergic reactions) were recorded.

Patients were excluded from the per‐protocol (PP) analysis if they were converted to general anesthesia (GA), received preoperative opioid medication, required additional perioperative sedation or blood transfusion, were not admitted to PACU, or if the operative duration exceeded 4 h.

### 2.6. Outcomes Measurement

The primary outcome was the incidence of POS (shivering score ≥ 1 at any perioperative time point). Secondary outcomes were incidence of hypothermia, shivering scores, perioperative core body temperature, frequency and total dose of pethidine administered for shivering, and the occurrence of adverse perioperative events (hypothermia, nausea and vomiting, and allergic reactions).

### 2.7. The Sample Size Calculation

Sample size was calculated using “N4Studies” (binary outcome RCT formula), referencing previous findings from Khalili et al. [19], which reported shivering incidences of 15.6% with paracetamol and 43.8% in controls. With *α* = 0.05% and 80% power, a minimum of 41 patients per group was necessary. Allowing for a 10% dropout rate, 46 patients were recruited per group.

### 2.8. Statistical Analysis

All data were analyzed by an independent statistician using STATA 18.0 (StataCorp, College Station, TX, USA). The primary outcome (incidence of POS) was evaluated using a PP analysis. Patients with protocol deviations, including conversion to GA, unprotocolled sedatives or opioids, blood transfusion, or operative duration exceeding 4 h, were excluded from the PP analysis. Because BSAS is invalid under GA, true intention‐to‐treat (ITT) assessment was not feasible for patients converted to GA. Therefore, to test the robustness of our PP findings in response to peer review, we conducted post hoc bounding sensitivity analyses for all six excluded patients: (1) a best‐case scenario (assuming all missing paracetamol patients did not shiver and all missing control patients shivered) and (2) a worst‐case scenario (assuming all missing paracetamol patients shivered and all missing control patients did not shiver). A post hoc fragility index was also calculated for the primary outcome to assess the robustness of the statistical significance.

Continuous variables were assessed for normality by the Shapiro–Wilk test. Normally distributed variables were expressed as mean ± standard deviation (SD) and compared using Student′s *t*‐test. Nonnormally distributed variables were reported as median (interquartile range, IQR) and analyzed with the Mann–Whitney *U* test. Categorical variables were presented as number (percentages) and compared by Fisher’s exact or Chi‐square test, as appropriate. A two‐sided *p* value < 0.05 was considered statistically significant.

For perioperative body temperature, between‐group comparisons were performed separately at each recorded intraoperative and PACU time point. To explore the relationship between temperature and shivering severity, Spearman’s rank correlation was used to assess the association between the lowest recorded body temperature and the highest BSAS score. In addition, binary logistic regression was performed to evaluate whether the lowest body temperature predicted the occurrence of POS, adjusting for group assignment.

## 3. Results

A total of 115 patients were assessed for eligibility. Of these, 23 patients were excluded prior to randomization (18 did not meet the inclusion criteria and 5 declined to participate). The remaining 92 patients were randomized equally to either the paracetamol group (*n* = 46) or the control group (*n* = 46), and all received their allocated interventions. Postrandomization, 6 patients were excluded from the PP analysis. In the paracetamol group, 4 patients were discontinued (3 required conversion to GA, and 1 had an operative time exceeding 4 h). In the control group, 2 patients were discontinued due to conversion to GA. There were no losses to follow‐up in either group. Ultimately, 42 patients in the paracetamol group and 44 patients in the control group were included in the final statistical analysis (Figure 1).

**FIGURE 1:**
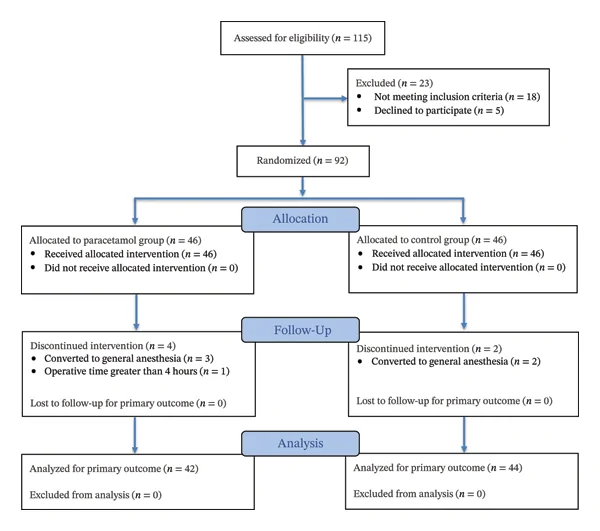
CONSORT diagram of the study population.

A total of 86 patients were included in the final analysis. Overall, the study population consisted of an equal proportion of males and females. The majority of the patients were classified as ASA physical status I and II, accounting for 60.5% and 36.0% of the cohort, respectively. Regarding the surgical specialties, orthopedic procedures were the most common (75.6%), followed by gynecologic (15.1%) and general surgeries (9.3%).

When comparing the study assignments, patient demographics, and baseline clinical parameters—including age, sex, weight, body mass index (BMI), ASA classification, and baseline body temperature—were well balanced with no statistically significant differences between the groups. Similarly, intraoperative characteristics, including the distribution of surgical types, duration of surgery, intraoperative morphine, administered intrathecal bupivacaine doses, and the achieved level of sensory blockade (ranging from T4 to T12 dermatomes), were entirely comparable between the two groups (Table 1).

**TABLE 1:** Baseline and intraoperative characteristics of patients undergoing spinal anesthesia by shivering prevention group.

Variables	Paracetamol group (*n* = 42)	Control group (*n* = 44)	*p* value
Age (years); mean ± SD^a^	46.0 ± 15.9	44.8 ± 15.7	0.735
Male sex; *n* (%)^c^	23 (54.8)	20 (45.5)	0.388
Weight (kg); mean ± SD^a^	68.0 ± 15.5	65.4 ± 13.0	0.405
BMI (kg/m^2^); mean ± SD^a^	24.9 ± 4.9	24.6 ± 4.5	0.784
ASA classification; *n* (%)^c^			0.159
I	23 (54.8%)	29 (65.9%)	
II	16 (38.1%)	15 (34.1%)	
III	3 (7.1%)	0 (0)	
Type of surgery; *n* (%)^c^			0.718
Orthopedics	33 (78.6)	32 (72.7)	
Gynecology	5 (11.9)	8 (18.2)	
General surgery	4 (9.5)	4 (9.1)	
Baseline temperature; mean ± SD^a^	36.6 ± 0.4	36.7 ± 0.3	0.659
Duration of surgery (minutes); mean ± SD^a^	133.1 ± 55.1	129.8 ± 51.5	0.773
Intraoperative morphine used; *n* (%)^b^	2 (4.8)	0 (0)	0.236
Intrathecal bupivacaine (mg); mean ± SD^a^	14.5 ± 1.6	14.4 ± 1.4	0.738
Level of sensory blockage; *n* (%)^c^			0.477
T4	4 (9.5)	5 (11.4)	
T5	1 (2.4)	3 (6.8)	
T6	14 (33.3)	13 (29.5)	
T7	1 (2.4)	1 (2.3)	
T8	5 (11.9)	8 (18.2)	
T10	17 (40.5)	12 (27.3)	
T12	0 (0)	2 (4.5)	

*Note:* Data were compared using ^a^Student’s t‐test; ^b^Fisher’s exact test; ^c^Pearson chi‐square test.

Abbreviations: ASA, American Society of Anesthesiologists; BMI, Body Mass Index; SD, standard deviation; T, thoracic.

The primary outcomes regarding POS are summarized in Table 2. In the PP analysis, the overall POS occurred in 11 of 42 patients (26.2%) in the paracetamol group compared to 22 of 44 patients (50.0%) in the control group (RR: 0.52, 95% CI: 0.29–0.94; *p* = 0.023). The absolute risk reduction (ARR) was 23.8% (95% CI, 4.2%–41.4%), and the number needed to treat (NNT) was 4.2 (95% CI, 2.4–23.8), indicating that approximately 4 patients would need to receive prophylactic IV paracetamol to prevent one additional case of POS. To address the ITT principle, we performed post hoc sensitivity analyses encompassing all 92 randomized patients. Under the best‐case scenario, the significant protective effect of paracetamol was maintained (23.9% vs. 52.2%; RR 0.46, 95% CI 0.26–0.82; *p* = 0.005). Under the extreme worst‐case scenario, the incidence of POS was 32.6% in the paracetamol group versus 47.8% in the control group (RR 0.68, 95% CI 0.41–1.14; *p* = 0.137). Intraoperative shivering occurred in 2 patients (4.8%) in the paracetamol group and 10 patients (22.7%) in the control group (*p* = 0.016). However, given the small number of events, this subgroup finding should be interpreted as exploratory and hypothesis‐generating rather than as a primary conclusion. In the PACU, postoperative shivering occurred in 10 patients (23.8%) in the paracetamol group compared with 17 patients (38.6%) in the control group although the difference was not statistically significant (*p* = 0.139).

**TABLE 2:** Comparison of the incidence of perioperative shivering between the study groups.

Incidence of POS	Paracetamol group (*n* = 42)	Control group (*n* = 44)	RR (95% CI)	*p* value
*Primary outcome (per-protocol analysis)*				
Overall POS	11 (26.2)	22 (50.0)	0.52 (0.29–0.94)	0.023^∗^

*Secondary outcomes (timing of shivering)*				
Intraoperative	2 (4.8)	10 (22.7)	0.21 (0.05–0.90)	0.016^∗^
PACU	10 (23.8)	17 (38.6)	0.62 (0.32–1.19)	0.139

*Post hoc ITT sensitivity analyses (n = 46/group)*				
Best‐case scenario ITT	11 (23.9)	24 (52.2)	0.46 (0.26–0.82)	0.005^∗^
Worse‐case scenario ITT	15 (32.6)	22 (47.8)	0.68 (0.41–1.14)	0.137

*Note:* Data are presented as number (percentage); POS were compared using the Pearson chi‐square test; for overall POS, ARR was 23.8% (95% CI, 4.2% to 41.4%) and NNT was 4.2 (95% CI, 2.4 to 23.8).

Abbreviations: ARR, absolute risk reduction; ITT, intention‐to‐treat; NNT, number needed to treat; PACU, Postanesthesia Care Unit; POS, perioperative shivering.

^∗^ *p* < 0.05 was statistical significance.

Regarding perioperative thermal profiles, baseline core body temperatures were comparable between the two groups. As illustrated in Figure 2, intraoperative core body temperatures followed similar patterns over time after SA, with no statistically significant differences between the paracetamol and control groups at any 15‐min interval throughout surgery (*p* > 0.05 at all time points). Similarly, in the PACU (Figure 3), core body temperatures gradually increased in both groups, with no statistically significant differences between the groups at any time point (*p* > 0.05 at all time points).

**FIGURE 2:**
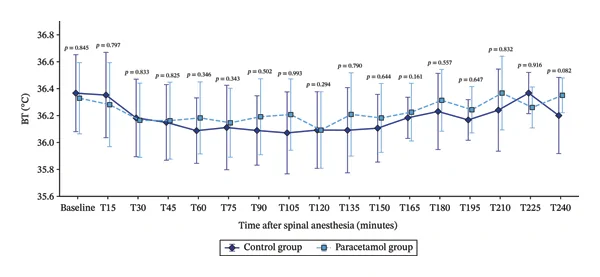
Comparison of intraoperative body temperature changes between groups. Data are expressed as mean ± SD. BT, body temperature; T, time after spinal anesthesia (minutes).

**FIGURE 3:**
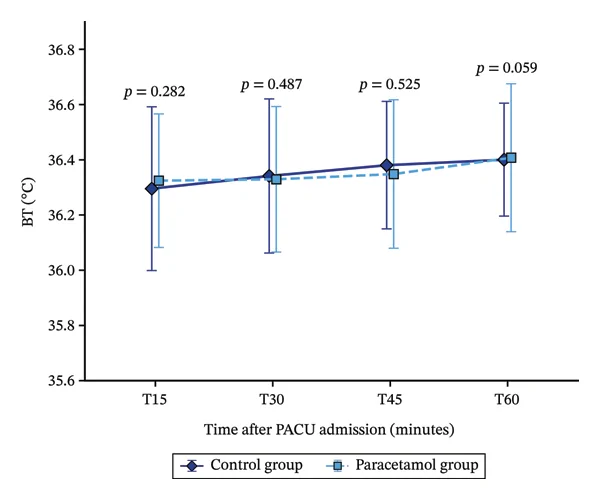
Comparison of temperature changes between groups in postanesthesia care unit. Data are expressed as mean ± SD. BT, body temperature; T, time after PACU admission (minutes); PACU, postanesthesia care unit.

To explore the relationship between body temperature and shivering, we performed Spearman correlation between the lowest body temperature and the highest BSAS score, which showed no significant association (*ρ* = 0.028, *p* = 0.800). Binary logistic regression with POS incidence as the outcome (adjusted for group) also demonstrated that lowest body temperature was not a significant predictor (OR = 0.71, 95% CI: 0.11–4.69; *p* = 0.721).

Regarding shivering severity and management, the paracetamol group demonstrated a significantly lower highest BSAS score, with a median of 0 (IQR 0–1) compared to 1 (IQR 0–2) in the control group (*p* = 0.039). The duration of shivering was also significantly shorter in the paracetamol group (*p* = 0.031). Consequently, both the patients requiring rescue pethidine and the pethidine dosage administered were significantly reduced in the paracetamol group (*p* = 0.019 and *p* = 0.023, respectively). Furthermore, there were no statistically significant differences regarding the incidence of hypothermia, nausea, and vomiting between the two groups. No allergic reactions were observed in either study group (Table 3).

**TABLE 3:** Comparison of shivering severity, rescue requirements, and adverse events between the study groups.

Variables	Paracetamol group (*n* = 42)	Control group (*n* = 44)	*p* value
Highest BSAS score; median (IQR)^a^	0 (0, 1)	1 (0, 2)	0.039^∗^
Shivering duration (minutes), median (IQR)^a^	0 (0, 2.8)	2.5 (0, 10)	0.031^∗^
Patients requiring rescue pethidine; *n* (%)^b^	6 (14.3)	16 (36.4)	0.019^∗^
Total rescue pethidine dose (mg): median (IQR)^a^	0 (0, 0)	0 (0, 12.5)	0.023^∗^
Hypothermia; *n* (%)^b^	20 (47.6)	21 (47.7)	0.992
Nausea and vomiting; *n* (%)^c^	4 (9.5)	3 (6.8)	0.710
Allergic reaction; *n* (%)^c^	0 (0)	0 (0)	NA

*Note:* Data were compared using ^a^Mann–Whitney U test; ^b^Pearson chi‐square test; ^c^Fisher’s exact test.

Abbreviations: BSAS, Bedside Shivering Assessment Scale; IQR, interquartile range; NA, not applicable.

^∗^ *p* < 0.05 was statistical significance.

## 4. Discussion

In this study, we demonstrated that the prophylactic administration of IV paracetamol significantly reduced the overall incidence of POS from 50.0% to 26.2% in patients undergoing SA. Furthermore, the paracetamol group exhibited lower shivering scores and duration and required significantly less rescue pethidine. These findings support the clinical utility of IV paracetamol not only in preventing POS but also in attenuating its severity when it does occur.

The precise mechanism underlying the antishivering effect of paracetamol remains incompletely understood and is likely multifactorial. Paracetamol has classically been proposed to act centrally by inhibiting cyclooxygenase‐mediated prostaglandin E2 (PGE2) synthesis in the hypothalamus, thereby modulating the thermoregulatory set‐point and reducing shivering [12]. However, more recent evidence suggests that paracetamol may also directly blunt thermogenic effector capacity, potentially affecting both shivering and non‐shivering thermogenesis independently of its central effects on the hypothalamic set‐point [24, 25]. Additionally, its favorable pharmacokinetic profile—achieving peak plasma concentrations within 15 min of IV administration and maintaining therapeutic levels for 4–6 hours—makes it appropriate for preventing post‐SA shivering in the perioperative setting.

The incidence of POS after neuraxial anesthesia in our control group was 50%, which is consistent with the prior reported range of 40%–64% [1, 2]. The significant reduction to 26.2% in the paracetamol group aligns with a recent meta‐analysis [20], which demonstrated a decrease in shivering from 52% to 22% with paracetamol. However, it should be noted that the studies included in the meta‐analysis—along with several recent randomized controlled trials—were predominantly conducted in patients undergoing GA. Consequently, these studies mainly reflect postoperative outcomes in the recovery room rather than the prophylactic effects on intraoperative shivering unique to regional anesthesia.

Among the recent trials evaluating IV paracetamol under GA, studies in obstetric populations have demonstrated that prophylactic paracetamol significantly reduces the incidence of shivering following cesarean delivery under GA from 50.9% to 9.1% [18]. Similarly, in nonobstetric GA settings—including upper limbs surgery, gynecological laparotomy, septoplasty, and inguinal hernia surgery—IV paracetamol has been shown to be an effective pre‐emptive strategy to mitigate postoperative shivering [19, 26–28]. While these prior studies strongly establish paracetamol’s antishivering efficacy following GA.

Unlike GA—which centrally suppresses the hypothalamus and typically triggers postoperative shivering during recovery—SA preserves hypothalamic function. Under SA, the primary thermoregulatory insult is redistribution hypothermia caused by sympathetic blockade‐induced vasodilation, often leading to intraoperative shivering due to core‐to‐periphery heat shunting, even without a major drop in core temperature [29]. This distinct pathophysiology highlights the need to evaluate antishivering treatments specifically within the SA context.

Our findings are supported by a recent trial evaluating paracetamol under SA. For instance, Pujitha et al. [30] demonstrated that IV paracetamol (15 mg/kg) administered 15 min after SA was associated with a lower incidence of severe shivering in the paracetamol group than that in the placebo group (10% vs. 70%, respectively; *p* < 0.001) and lower overall shivering scores. Similarly, Esmat et al. [12] reported that prophylactic oral paracetamol (1 g) significantly decreased clinically significant postspinal shivering from 77% to 15% (*p* < 0.001) in patients undergoing lower abdominal and lower limb surgeries.

Shivering in patients undergoing cesarean section under SA has also been reported. For instance, Prasetyo et al. [31] investigated prophylactic IV paracetamol (15 mg/kg) in cesarean section patients and reported a significantly lower incidence of shivering (35.3% vs. 76.5%, *p* = 0.038) along with a delayed onset time compared to controls. These results align closely with Rasoli et al. [21], who demonstrated that prophylactic IV paracetamol (1 g) effectively attenuated the mean increase in shivering scores in a similar obstetric cohort (*p* = 0.041). Interestingly, while Prasetyo et al. [31] observed no significant reduction in interleukin‐6 (IL‐6) levels—suggesting that shivering mitigation may not be strictly tied to the suppression of systemic inflammation in this specific context—the consistent clinical reduction in shivering incidence strongly reinforces the primary hypothesis.

However, our results contrast sharply with established literature when considering the influence of routine perioperative care. A recent RCT by Ularangkura et al. [32] evaluating prophylactic paracetamol in cesarean section under SA also reported a null finding—no significant reduction in shivering incidence. The crucial difference appears to stem from superior active warming protocols employed in their setting; they maintained core temperature effectively via routine forced‐air warming blankets and prewarmed IV fluids, resulting in a remarkably low baseline shivering incidence in the control group (15%) [32]. These divergent results highlight an important contextual consideration: in centers where intensive active warming achieves a very low baseline shivering rate, the incremental antishivering benefit of paracetamol may be reduced and more difficult to demonstrate. However, in the present trial, despite standardized forced‐air warming and prewarmed IV fluids, the control group shivering rate remained high (50%), leaving a substantial residual risk that paracetamol significantly reduced. Therefore, paracetamol may still provide meaningful benefit in settings where routine active warming alone is insufficient to prevent POS; this hypothesis warrants confirmation in future studies incorporating active warming comparators or standard‐care reference arms.

A notable and physiologically important observation in this trial is the dissociation between thermoregulatory outcomes. The incidence of intraoperative hypothermia (47.6% vs. 47.7%, *p* = 0.992). Consistent with our findings, previous studies have reported that although core body temperature decreased significantly following SA, the magnitude of temperature reduction did not differ significantly between patients receiving paracetamol and those receiving placebo or control treatment [12, 18, 30]. Our additional correlation and regression analyses further supported this observation. Spearman’s rank correlation demonstrated no statistically significant relationship between the lowest core body temperature and the highest BSAS score. Similarly, binary logistic regression confirmed that the lowest core temperature was not a significant predictor of shivering occurrence. These results suggest that shivering after SA may be influenced by factors beyond changes in core temperature, including internal heat redistribution and peripheral thermal inputs.

The comparable temperature profiles despite differences in POS incidence suggest that the antishivering effect of paracetamol may not primarily result from the prevention of heat loss or the maintenance of core temperature. Instead, this finding is consistent with a potential central thermoregulatory mechanism. Paracetamol may inhibit cyclooxygenase‐mediated prostaglandin synthesis in the hypothalamus and modulate the thermoregulatory set point, thereby reducing the propensity for shivering without substantially altering core temperature [33]. However, this proposed mechanism requires further investigation.

Crucially, this temperature profile contrasts with findings under GA. In trials like that by Khalili et al. [19], paracetamol administration led to significantly greater core temperature reductions in the treated group. This divergence stems from the mechanism of anesthesia: SA leaves some compensatory thermoregulatory zones active, whereas GA induces profound, systemic vasodilation, amplifying the physiological impact of the lowered hypothalamic set‐point and resulting in more pronounced hypothermia.

Our analysis further revealed a clinically significant lower pethidine requirement, with 14.3% in the paracetamol versus 36.4% in the control group for shivering treatment. This represents an important clinical advantage of paracetamol by minimizing opioid exposure and its associated complications, including nausea, vomiting, and respiratory depression.

### 4.1. Limitations

This study has several limitations. First, PP analysis was utilized as the primary analytic approach, while ITT bounding sensitivity analyses (best‐case and worst‐case scenarios) were conducted post hoc. A true ITT analysis was precluded because 5 of the 6 excluded patients underwent intraoperative conversion to GA—a state in which the BSAS cannot be validly administered. We report these post hoc bounding sensitivity analyses transparently so that readers may evaluate the findings under extreme assumptions. Second, our primary outcome yielded a post hoc fragility index of 2, indicating that the observed statistical significance is sensitive to small event changes. Third, blinding success was not formally assessed, leaving a potential risk of residual unblinding. Fourth, detailed intraoperative fluid volumes and blood transfusion data were not systematically recorded although standardized thermal protocols were uniformly applied. Fifth, continuous time‐to‐event data (such as time to the first shivering episode) were not recorded, limiting detailed survival or onset‐timing analyses. Finally, the placebo‐controlled design precluded direct comparison with an active warming comparator or with other antishivering agents; therefore, the incremental utility of paracetamol relative to optimized active warming or established pharmacological therapies remains uncertain. Future trials with larger sample sizes should prespecify contingency strategies for outcome ascertainment when clinical assessment scales may be affected by changes in anesthetic state, while further evaluating elderly populations, relevant clinical variables, and optimal IV paracetamol regimens.

## 5. Conclusion

In patients undergoing surgery under SA, prophylactic IV paracetamol when added to standardized active warming reduced the incidence, severity, and duration of POS and lowered the requirement for rescue pethidine compared with placebo. Further studies with active comparator or standard‐care reference arms are warranted to define its incremental role within multimodal normothermia strategies.

## Author Contributions

Conceptualization: all authors.

Formal analysis: Thitinuch Ruenhunsa, Saranyoo Nonphiaraj, and Donlaya Srikajonkiat.

Funding acquisition: Thitinuch Ruenhunsa, Saranyoo Nonphiaraj, and Donlaya Srikajonkiat.

Methodology: all authors.

Visualization: all authors.

Writing–original draft preparation: Thitinuch Ruenhunsa and Donlaya Srikajonkiat

Writing–review and editing: Saranyoo Nonphiaraj and Sarinya Chanthawong

## Funding

The authors gratefully acknowledge financial support from the Faculty of Medicine, Khon Kaen University, Thailand (Grant number IN64211).

## Ethics Statement

The present trial was approved by the Center for Ethics in Human Research, Khon Kaen University (Reference No: HE631446). The trial was conducted in accordance with the Declaration of Helsinki and Good Clinical Practice guidelines. Written informed consent was obtained from every participant prior to enrollment.

## Conflicts of Interest

The authors declare no conflicts of interest.

## Data Availability

The datasets generated and analyzed during the present study are available from the corresponding author upon reasonable request.

## References

[1] Crowley L. and Buggy D. , Shivering and Neuraxial Anesthesia, Regional Anesthesia and Pain Medicine. (2008) 33, no. 3, 241–252, 10.1016/j.rapm.2007.11.006. 18433676

[2] Usta B. , Gözdemir M. , Demircioglu R. I. , Muslu B. , Sert H. , and Yaldız A. , Dexmedetomidine for the Prevention of Shivering During Spinal Anesthesia, Clinics. (2011) 66, no. 7, 1187–1191, 10.1590/S1807-59322011000700011. PMC3148462 21876972

[3] Jain A. , Gray M. , Slisz S. , Haymore J. , Badjatia N. , and Kulstad E. , Shivering Treatments for Targeted Temperature Management: A Review, Journal of Neuroscience Nursing. (2018) 50, no. 2, 63–67, 10.1097/JNN.0000000000000340. PMC5882245 29278601

[4] Ozaki M. , Kurz A. , and Sessler D. I. , Thermoregulatory Thresholds During Epidural and Spinal Anesthesia, Anesthesiology. (1994) 81, no. 2, 282–288, 10.1097/00000542-199408000-00004. 8053576

[5] Sessler D. I. , Warner D. S. , and Warner M. A. , Temperature Monitoring and Perioperative Thermoregulation, Anesthesiology. (2008) 109, no. 2, 318–338, 10.1097/ALN.0b013e31817f6d76. PMC2614355 18648241

[6] Sessler D. I. , Postanaesthetic Shivering–From Pathophysiology to Prevention, Romanian Journal of Anaesthesia and Intensive Care. (2018) 25, no. 1, 10.21454/rjaic.7518.251.xum. PMC5931188 29756066

[7] Zhang J. , Zhang X. , Wang H. , Zhou H. , Tian T. , and Wu A. , Dexmedetomidine as a Neuraxial Adjuvant for Prevention of Perioperative Shivering: Meta-Analysis of Randomized Controlled Trials, Public Library of Science ONE. (2017) 12, no. 8, 10.1371/journal.pone.0183154. PMC5567500 28829798

[8] Amsalu H. , Zemedkun A. , Regasa T. , and Adamu Y. , Evidence-Based Guideline on Prevention and Management of Shivering After Spinal Anesthesia in Resource-Limited Settings: Review Article, International Journal of General Medicine. (2022) 15, 6985–6998, 10.2147/IJGM.S370439. PMC9462549 36090703

[9] Rauch S. , Miller C. , Bräuer A. , Wallner B. , Bock M. , and Paal P. , Perioperative Hypothermia—A Narrative Review, International Journal of Environmental Research and Public Health. (2021) 18, no. 16, 10.3390/ijerph18168749. PMC8394549 34444504

[10] Plengpanich P. , Charoensuk C. , Chanthawong S. et al., Effect of Short Duration Used of Active Prewarming for Preventing Hypothermia in Non-Anesthetized Volunteer: A Randomized-Controlled Trial, Journal of the Medical Association of Thailand. (2023) 106, no. 1, 95–101, 10.35755/jmedassocthai.2023.01.13747.

[11] Joo Y. , Kim H. J. , Kim J. T. et al., Effect of Active Warming on Shivering During Spinal Anesthesia, Korean Journal of Anesthesiology. (2009) 57, no. 2, 176–181, 10.4097/kjae.2009.57.2.176. 30625853

[12] Esmat I. M. , Mohamed M. M. , Abdelaal W. A. , El-Hariri H. M. , and Ashoor T. M. , Postspinal Anesthesia Shivering in Lower Abdominal and Lower Limb Surgeries: A Randomized Controlled Comparison Between Paracetamol and Dexamethasone, BioMed Central Anesthesiology. (2021) 21, no. 1, 10.1186/s12871-021-01483-7. PMC8556952 34717535

[13] Mahesh T. , A Randomised Trial Comparing Efficacy, Onset and Duration of Action of Pethidine and Tramadol in Abolition of Shivering in the Intra Operative Period, Journal of Clinical and Diagnostic Research. (2014) 8, 10.7860/JCDR/2014/10584.5148. PMC4290338 25584238

[14] Reddy V. and Chiruvella S. , Clonidine Versus Tramadol for Post Spinal Shivering During Caesarean Section: A Randomized Double Blind Clinical Study, Journal of Obstetric Anaesthesia and Critical Care. (2011) 1, no. 1, 26–29, 10.4103/2249-4472.84252.

[15] Kim Y. A. , Kweon T. D. , Kim M. , Lee H. I. , Lee Y. J. , and Lee K. Y. , Comparison of Meperidine and Nefopam for Prevention of Shivering During Spinal Anesthesia, Korean Journal of Anesthesiology. (2013) 64, no. 3, 229–234, 10.4097/kjae.2013.64.3.229. PMC3611072 23560188

[16] Hameed M. , Ali N. , Ahsan K. , and Nazir M. , Pharmacological Interventions for the Treatment and Control of Shivering in Adult Patients Undergoing Elective Surgery Under Regional Anaesthesia: A Systematic Review and Meta-Analysis, Turkish Journal of Anaesthesiology and Reanimation. (2022) 50, no. 4, 246–254, 10.5152/tjar.2021.20008. PMC9524425 35979970

[17] Mohta M. , Mounika T. B. , and Tyagi A. , Effect of Timing of Intraoperative Administration of Paracetamol on Postoperative Shivering: A Randomised Double-Blind Controlled Trial, Indian Journal of Anaesthesia. (2023) 67, no. 12, 1071–1076, 10.4103/ija.ija_720_23. PMC10858702 38343687

[18] Gholami A. and Hadavi M. , Prophylactic Intravenous Paracetamol for Prevention of Shivering After General Anesthesia in Elective Cesarean Section, Journal of Obstetric Anaesthesia and Critical Care. (2016) 6, no. 2, 81–85, 10.4103/2249-4472.191601.

[19] Khalili G. , Sajedi P. , and Alinaghian A. , The Effect of Intravenous Infusion of Paracetamol Before Anesthesia Induction on the Core and Peripheral Temperature Changes and Post-Operative Shivering in Patients Undergoing General Anesthesia, Advanced Biomedical Research. (2014) 3, no. 1, 10.4103/2277-9175.128468. PMC3988594 24761397

[20] Liu J. , Cao Q. , Zeng J. , and Liang X. , Efficacy of Intravenous Acetaminophen on Postoperative Shivering: A Meta-Analysis of Randomized Controlled Trials, Medicine (Baltimore). (2024) 103, no. 28, 10.1097/MD.0000000000038710. PMC11245272 38996153

[21] Rasoli S. , Ansari E. , Moslemi F. , and Ghojazadeh M. , The Prophylactic Administration of Intravenous Paracetamol for Control of Shivering During and After Cesarean Section Under Spinal Anesthesia, Ayush Admissions Central Counseling Committee. (2019) 5, 10.18502/aacc.v5i2.748.

[22] Badjatia N. , Strongilis E. , Gordon E. et al., Metabolic Impact of Shivering During Therapeutic Temperature Modulation: The Bedside Shivering Assessment Scale, Stroke. (2008) 39, no. 12, 3242–3247, 10.1161/STROKEAHA.108.523654. 18927450

[23] Olson D. M. , Grissom J. L. , Williamson R. A. , Bennett S. N. , Bellows S. T. , and James M. L. , Interrater Reliability of the Bedside Shivering Assessment Scale, American Journal of Critical Care. (2013) 22, no. 1, 70–75, 10.4037/ajcc2013907. 23283091

[24] Esh C. J. , Chrismas B. C. R. , Mauger A. R. , and Taylor L. , Pharmacological Hypotheses: Is Acetaminophen Selective in Its Cyclooxygenase Inhibition?, Pharmacology Research & Perspectives. (2021) 9, no. 4, 10.1002/prp2.835. PMC8287062 34278737

[25] Foster J. , Mauger A. , Thomasson K. , White S. , and Taylor L. , Effect of Acetaminophen Ingestion on Thermoregulation of Normothermic, Non-Febrile Humans, Frontiers in Pharmacology. (2016) 7, 10.3389/fphar.2016.00054. PMC4789353 27014068

[26] Kinjo T. , Tadokoro T. , Tokushige A. et al., Effects of Perioperative Administration of Acetaminophen on Postoperative Shivering: A Randomized, Triple-Blind, Placebo-Controlled Trial, Anesthesia & Analgesia. (2020) 130, no. 4, 983–990, 10.1213/ANE.0000000000004306. 31274602

[27] Kashif S. , Kundi M. N. , and Khan T. A. , Pre-Emptive Effect of Intravenous Paracetamol Versus Intravenous Ketorolac on Post-Operative Pain and Shivering After Septoplasty Under General Anesthesia: A Comparative Study, Pakistan Armed Forces Medical Journal. (2021) 71, no. 4, 1179–1182, 10.51253/pafmj.v71i4.4269.

[28] Karbord A. , Effectiveness Dose of Injection Acetaminophen for Control of Shivering After General Anesthesia in Inguinal Hernia Surgery, Biomedical Journal of Scientific & Technical Research. (2023) 49, no. 3, 10.26717/BJSTR.2023.49.007791.

[29] Sessler D. I. , Perioperative Thermoregulation and Heat Balance, Lancet. (2016) 387, no. 10038, 2655–2664, 10.1016/S0140-6736(15)00981-2. 26775126

[30] Pujitha K. N. S. , Prasad U. S. S. A. V. , Sudhakar B. , and Pradeep K. S. D. , Effects of Perioperative Administration of Acetaminophen on Postoperative Shivering, International Journal of Current Science and Pharmaceutical Research. (2025) 15, no. 3, 10.56975/ijcsp.v15i3.303125.

[31] Prasetyo A. I. , Husain A. A. , Musba A. M. T. , Salam S. H. , and Ratnawati W. T. C. , The Effect of Paracetamol Administration on Interleukin-6 Levels and the Incidence of Shivering in Cesarean Section Patients With Spinal Anesthesia, Ayush Admissions Central Counseling Committee. (2025) 11, 10.18502/aacc.v11i5.19917.

[32] Ularangkura W. , Chokchalermwong N. , and Sukonpatip T. , Efficacy of Intravenous Paracetamol for Prevention of Shivering in Cesarean Section Under Spinal Anesthesia: A Randomized Controlled Trial, Thai Journal of Anesthesiology. (2023) 49, no. 5, 311–318.

[33] Mallet C. , Desmeules J. , Pegahi R. , and Eschalier A. , An Updated Review on the Metabolite (AM404)-Mediated Central Mechanism of Action of Paracetamol (Acetaminophen): Experimental Evidence and Potential Clinical Impact, Journal of Pain Research. (2023) 16, 1081–1094, 10.2147/JPR.S393809. PMC10066900 37016715

